# Ultrasound-Assisted Stable Curcumin Nanoemulsion and Its Application in Bakery Product

**DOI:** 10.1155/2022/4784794

**Published:** 2022-12-16

**Authors:** Uday Bagale, Ammar Kadi, Artem Malinin, Irina Potoroko, Shirish Sonawane, Shital Potdar

**Affiliations:** ^1^Department of Food and Biotechnology, South Ural State University, Chelyabinsk 454080, Russia; ^2^Department of Chemical Engineering, National Institute of Technology, Warangal 506004, India

## Abstract

The quality of the bread has been always an important issue and needs to be improved. Curcumin nanoemulsion provides an antioxidant and other nutritional value to the bakery products. Our aim was to determine the effect of curcumin nanoemulsions as a food additive on the quality and digestibility of breads. Curcumin nanoemulsion was stabilized by using Tween 80 and an ultrasound approach and its incorporation of curcumin nanoemulsion into bread formulation as the replacement of margarine. The objects of the study were the obtained bread from wheat flour, namely, control sample, CuNE containing sample, and raw curcumin containing bread sample. The results of the sensory evaluation of prototype bread suggest that curcumin nanoemulsion does affect organoleptic properties of bread. The result of antioxidant activity for curcumin nanoemulsion bread is higher (31.59%) compared to a control bread (20.59%). Also, in addition to a positive effect, there is an increase in the total strain and the elasticity of the crumb of bread compared to the control bread. SEM (scanning electron microscope) study shows that formulation with nanoemulsion promotes uniform distribution of fine pores (porosity).

## 1. Introduction

The bakery industry in Russia is one of the main sectors of the food industry and carries out vital work to produce bread and bakery products. Bakery products and bread are included in the list of basic foodstuffs. Daily bread consumption is from 150 to 500 gms per day per capita in different countries [1, 2]. Bread is the physiological source of digestible carbohydrates, vegetable protein, water-soluble vitamins, dietary fiber, and several macro- and microelements [1, 3, 4]. To date, much attention has been paid to the enrichment of bread with various functional additives to give it preventive and curative properties. Introduction of additives with antioxidant activity in the formulation of bakery products will effectively solve the problem of prevention and treatment of various diseases [5–8]. One of the representatives of the most common antioxidants is diferuloilmetan (curcumin), a part of the turmeric root [9–13]. This substance has polyphenols, characterized by more than one phenolic group per molecule. Turmeric contains a wide variety of vitamins and other valuable micro- and macromolecules which are beneficial to health. Today, curcumin derived from the turmeric root is a part of many medicines and biologically active complexes, along with its usage in the food industry as food spices and natural dye [5, 7–10].

In addition, the polyphenol curcumin has been shown to target numerous signaling molecules and also exhibit activity at the cellular level, supporting its numerous health benefits. It has been shown to help with the management of inflammatory and degenerative eye conditions, metabolic syndrome, pain, and inflammatory conditions. It has also been demonstrated to be advantageous for the kidneys. While there seem to be countless therapeutic advantages to taking curcumin supplements, the majority of these advantages are brought on by its anti-inflammatory and antioxidant properties. Despite being claimed to have anti-inflammatory and antioxidant properties, curcumin's poor bioavailability is one of the main issues with ingesting it alone. This appears to be primarily because of poor rapid absorption [9, 11, 12]. However, the solubility issue of curcumin limits its direct use in the bread products. In recent years, there has been an active progress in the development of curcumin encapsulation systems in water nanoemulsions [13–15]. Nanosize curcumin has better surface charge and surface area, higher hydrophobicity, better optical properties, and even higher antioxidant activity than native curcumin. Change in scaling of curcumin increases the water solubility and dispersibility to improving the antimicrobial activity of curcumin [16–18]. Practice of nanoemulsions is a tremendous technique castoff to integrate these bioactive complexes into foods [19–21]. Sharma et al. [22] reported that the Tween 20 base oil-in-water nanoemulsion encapsulated the curcumin in it. As particle size decreases, the optical transparency and stability of curcumin-encapsulated nanoemulsion increase and vice versa. Abbas et al. [23] reported the encapsulation of curcumin in nanoemulsion stabilized by OSA-modified starch. OSA starches were successfully used to prepare and stabilize ultrasonic-assisted nanoemulsions. Although nanoemulsions were produced at all levels, the best values for the formulation and process parameters were found to produce the smallest possible droplet size emulsions with the shortest sonication time and delivered power. Hong et al. [12] discussed about emulsion powder (TE-NEP) containing turmeric which showed small droplets (200 ± 1.36 nm) with a very low zeta potential (−64 ± 0.75 mV). The TE-NEP was found to be stable in the majority of the test conditions, only exhibiting a significant increase in droplet size at high ethanol concentrations (>10%) and a decrease in zeta potential at extremely acidic pH and high salt concentrations. For up to 14 days of storage, the retention rate of curcumin exhibited values above 89.2%.

The research gap is that, till now, no study has reported curcumin nanoemulsion incorporation in the bakery product to enhance its nutritional value. In this work, we aim to fabricate stable curcumin nanoemulsion using Tween 80 as emulsifier and ultrasound technique. Later, nanoemulsions will be characterized using particle size, encapsulation efficiency, and stability data. Subsequently, prepared nanoemulsion was incorporated in bread formulation. To check the best bread quality, further bread formulations were analyzed for sensory and physicochemical properties.

## 2. Materials and Methods

Curcumin, palm oil, Tween 80, wheat flour, sugar and margarine, and yeast, all materials were procured from the local market of Chelyabinsk city, Russian Federation. U-sonic ultrasound with 400 W of amplitude 80% power, the frequency at 22 ± 1.65 kHz with tip diameter 22 mm, was used in all experiments. Distilled water was used for the carried-out experiment.

### 2.1. Curcumin Nanoemulsion Preparation Using Ultrasound

Initially, as reported by Ahmed et al. [5], curcumin powder of concentration (0.75 and 0.15 g) was dissolved in the palm oil under the mechanical agitation at 50°C to prepare a lipid phase of an emulsion. Later, the aqueous phase was prepared by dissolving Tween 80 of concentration (0.2 and 0.6 g) in distilled water.

Both aqueous and oil phases were mixed and emulsified together using high-intensity (80% amplitude) sonication operated at 22 kHz frequency and 400 W power of the ultrasonic processor. During the emulsification process, the temperature was monitored using a digital thermometer and maintained below 50°C. To minimize the hot spot generation during sonication, the total sonication time was split into 4 cycles (3 min/cycle) with the provision for jacketed cool water surrounding the reactor.

### 2.2. Physicochemical Analysis of Nanoemulsions

#### 2.2.1. Nanoemulsion Stability

Nanoemulsion stability was measured according to the method described by Kumar et al. [24]. The nanoemulsions were kept in a hot water bath at 80°C for 30 minutes and then transferred to an ice bath for 15 minutes, followed by centrifugation (Hettich Zentrifugen Mikro 22R) at 5000 rpm for 30 min. The whole volume (WV) of the nanoemulsion in the centrifuge tube and the volume of the nanoemulsion phase (EPV) were measured. The following formula calculated the nanoemulsion stability (ES):

(1) $$\begin{array}{c} \text{Nanomulsion stability }\left(\%\right)=\frac{\text{volume of nanoemulsion phase}}{\text{total volume of nanoemulsion}}\times 100. \end{array}$$

#### 2.2.2. Determination of pH, Particle Size Distribution, and Encapsulation Efficiency

An electrochemically assembled pH meter was used to measure the pH of the prepared nanoemulsions. A Nanotrac FLEX particle size analyzer evaluated the particle size distribution through the DLS method. The nanoemulsions were diluted with distilled water (1 : 100) for homogenous particle suspension in the particle size analysis. This suspension was mounted on Nanotrac external probe where light scattered coming from the sample is used to measure the particle size distribution.

Encapsulation efficiency is frequently used to describe the encapsulation of bioactive compounds. With some modification, encapsulation efficiency was calculated using the method of Surassmo et al. [25] 15 ml of prepared nanoemulsion was passed through the filter membrane cap and centrifuged at 5000 rpm and 5°C for 30 mins. After centrifuge permeate was collected, the UV (Shimadzu UV-2700, Japan) absorbance was recorded at 520 nm wavelength. All measurements were done in triplets.

#### 2.2.3. Preparation of Bread

As reported by Naumenko et al. [26], we baked three different prototypes of tin bread using formulations created in the lab to accomplish this goal (Table 1): control: flour-based white bread; sample 1 is bread made with dried, pulverized turmeric root; and sample 2 is bread made with a curcumin nanoemulsion. The developed formulation called for dissolving the pressed yeast in a small amount of water. Unorthodox methods were used to prepare the dough. According to the established recipes, pressed yeast and other ingredients are added. The melted margarine or nanoemulsion or ground turmeric root powder and the necessary quantity of presifted flour are also added. Bowl was capped and secured and the process was started. The duration of kneading was 30 minutes. The substituted dough was cut, for tin bread, and put on proofing in a thermostat. The whole process was kept at 60-70°C for two and half hours.

### 2.3. Analysis of Prepared Baked Bread

#### 2.3.1. Sensory Analysis of Bread

Taking into account the requirement of the SUSU (South Ural State University) Ethics Committee, we did sensory analysis of bread. To investigate the effect of dry crushed root of turmeric and nanoemulsion organoleptic quality indicators, the baked bread prototypes were held. To reduce the influence of personality characteristics of consumers, we used a variant ranking point. For the sensory evaluation of bread quality, a 5-point scale method was used (5-maximum and 0-worst). To establish different stages, scales have been developed to describe monitored characteristic gradations. This range takes into account the standard and optional organoleptic quality indicators [27].

### 2.4. Humidity, Acidity, and Porosity Analysis of Bread

#### 2.4.1. Humidity

According to the normative document GOST 21094-75 for bread and bakery products, the humidity indicator is determined.

As mentioned by Naumenko et al. [26], bread sample was cut into two equal pieces. The prepared sample is quickly and thoroughly crushed by mechanical shredder and then immediately weighed. The samples were preheated and kept at 130°C for 40 mins, then placed in a desiccator, allowed to cool, and then calibrated with an error of no more than 0.05 g.

Humidity (*W*) as a percentage is calculated using the following formula:

(2) $$\begin{array}{c} W=\frac{\left(m_{1}-m_{2}\right)}{m}\cdot 100, \end{array}$$

where *m*_1_ is the weight of the cup with the hitch before drying in g, *m*_2_ is the weight of the cup with a hitch after drying in g, and *m* is the weight of the product hitch (gm).

#### 2.4.2. Acidity Analysis of Bread Crumb

25 g of the bread crumbs is filled with 25% (250 ml) of the necessary distilled water, and the crumbs are quickly mixed to form a homogeneous mass. Later, the remaining 75% water was added to the mixture and shaken vigorously for 2 min. The liquid layer is then carefully drained into a beaker using a frequent sieve or gauze. A different solution is titrated with 2–3 drops of phenolphthalein against 0.1 M NaOH until the pink color disappears. Acidity can be calculated as

(3) $$\begin{array}{c} X=\frac{V\cdot V_{1}\cdot a}{10m\cdot V_{2}}\cdot K, \end{array}$$

where *V* is the volume of a solution of NaOH required ml, *V*_1_ is the volume of distilled water taken, *a* is the conversion factor for 100 g of sample, *K* is the correction factor maintaining 0.1 mol/l concentration of NaOH, 1/10 is the coefficient of reduction for NaOH, *m* is the weight of the hitch in g, and *V*_2_ is the volume of the test solution taken for titration in cm^3^.

#### 2.4.3. Porosity Analysis of Bread Crumb

As per norms of Russian documnet GOST 5669-96, the anlaysis of bakery product in terms of breas crumbs porosiy calculated as mensioned by Naumenko et al. [2]. The crumb-filled cylinder is placed on the tray slot. The bread crumb is pushed out of the cylinder until it measures 1 cm and cut off. The remaining crumb in the cylinder is pushed out to the tray wall and cut off at the cylinder edge. Make three cylindrical recess breads from a flour mixture—four recesses with a volume of 27 ± 0.5 ml each—to determine the porosity of wheat bread.

Porosity *П*, %, is calculated by the following formula:

(4) $$\begin{array}{c} \Pi =\frac{V-\left(m/\rho \right)}{V}\cdot 100, \end{array}$$

where *V* is the total volume of bread recesses in cm^3^, *m* is a mass of recesses in g, and *ρ* is the density of the nonporous mass of the crumb.

#### 2.4.4. Scanning Electron Microscope of Bread Samples

As mentioned by Naumenko et al. [26], for the study of colloidal and biochemical processes, bread crumb was analyzed by scanning electron microscopy (SEM). Bread crumb sample preparation for the study includes the chemical fixation of liquid CO_2_ under pressure. The samples were sputtered in thin gold layer with thickness of 2-5 nm by sputtering in an apparatus for localization signal on the sample surface and to enhance the conductivity.

#### 2.4.5. Deformation Dough Characteristics

Deformation test, crumb characteristics, and test samples of bread were determined on “Strukturometr ST-2.” As reported by Naumenko et al. [28], slices up to 25 mm thick were obtained from the bread samples (4 hours after baking). The crust layer was removed after the slices were cut to 25 mm long by 25 mm wide. An aluminum cylindrical probe P/20 (radius 20 mm) was used for the analysis, with the following experimental parameters: preliminary testing of 1.0 mm s^−1^, test of 1.7 mm s^−1^, reverse stroke of 10.0 mm s^−1^, and compressive force of 40%.

#### 2.4.6. Antioxidant Activity of Bread Samples

For the antioxidant activity of bread sample containing curcumin nanoemulsion, 0.1 mM DPPH radical solution was prepared. Using UV spectrophotometer at 515 nm, wavelength absorbance of solution was noted. Preparation of bread samples was consisted of 10 grams of the crumb in 90% ethanol at LOIP LS-120 laboratory shaker for 45 minutes at 150 rpm and subsequent centrifugation for 10 minutes, and the supernatant was taken for analysis. 20 *μ*l of supernatant was placed in a microplate, and 280 *μ*l of DPPH radical solution was added to each sample. Samples were incubated for 30 min in the dark, and the absorbance was measured against a wavelength of 517 nm [29].

(5) $$\begin{array}{c} \text{DPPH scavenging activity}\left(\%\right)=\frac{\text{Abs control}‐\text{Abs sample}}{\text{Abs control}}\times 100. \end{array}$$

For antioxidant activity with 2,2′-azino-bis(3-ethylbenzothiazoline-6-sulfonic acid) diammonium salt (ABTS), 30 *μ*l of the nanoemulsions was mixed with 3 ml of ABTS for sample preparation. The samples were analyzed with a spectrophotometer at 734 nm and kept in the dark for 24 minutes to react at 6 min intervals to accompany the absorbance reduction. The absorbance variation was measured, and the percentage inhibition was calculated using Equation (5).

#### 2.4.7. Microbial Analysis of Bread Sample

For microbial analysis, our main aim is to check the microbial growth on bread samples (control bread and with curcumin nanoemulsion bread). We have taken fresh bread sample slice in Petri dish and added external green algae which are already formed on stored bread samples. Later, samples were kept at two different conditions ((a) at room temperature and (b) at 4°C in refrigerator), and the sample was checked after 7 and 14 days for microbial growth [30].

## 3. Statistical Analysis

The data are expressed as mean ± standard error of mean (SEM) for each group. Statistical analysis was done using the GraphPad Prism version 8.0 software (GraphPad software, 2019). The value in the columns (Tables 2–5) is with standard deviation (*p* ≤ 0.05).

## 4. Result and Discussions

In the current investigation, initially we stabilize the curcumin nanoemulsion containing edible palm oil and Tween 80 by using Tween 80 along with a sonochemical approach. To get stable curcumin nanoemulsion (CuNE), we have optimized the oil and emulsifier concentration along with the curcumin concentration. In the current o/w nanoemulsion system, the curcumin concentration inner oil phase ranging from 0.75 g to 0.15 g was encapsulated in edible palm oil. We have tried different inner and outer phase combinations to make a stable nanoemulsion, but only a few were found to be stable on centrifuge and heating at 80°C for 30 min, as shown in Table 6.

### 4.1. Characterization of Nanoemulsion

The stable nanoemulsions were further studied for physiochemical analysis and their incorporation in bread formulation. CuNE physical properties such as particle size distribution, pH, and stability were carried out using an optical microscope, whereas structural property was carried out. The value of stable nanoemulsions with stability and pH is shown in Table 2. The gravitational stability of nanoemulsions against droplet aggregation was carried out by keeping them at room temperature.

### 4.2. Particle Size Distribution, Polydispersity Index (PDI), and Optical Microscope for Stable Nanoemulsion

**Figure 1:**
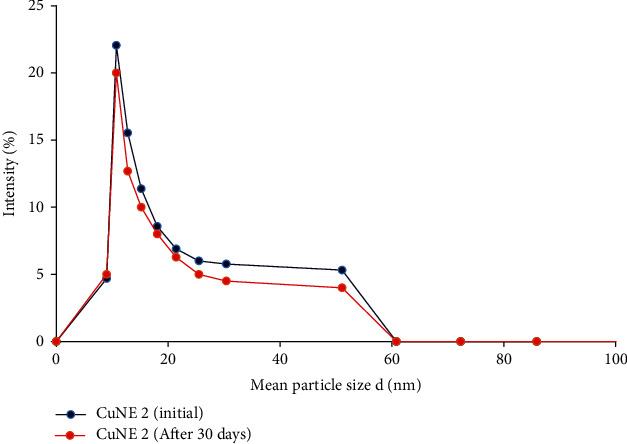
Stability data of nanoemulsion sample in term of PSD.

**Figure 2:**
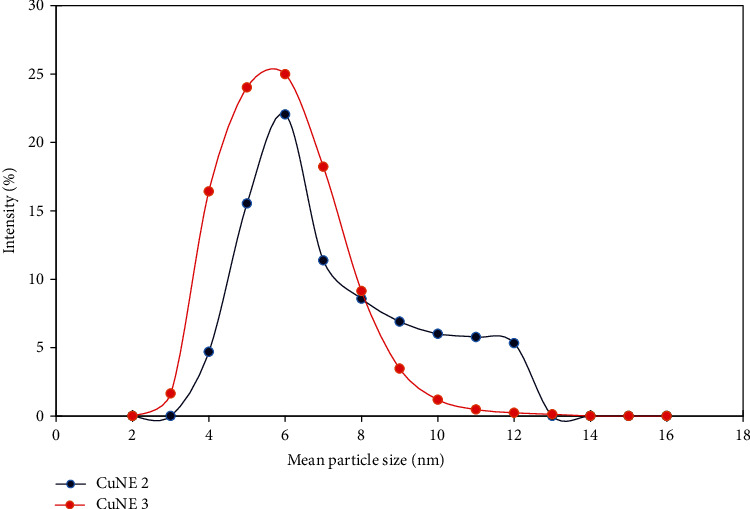
Particle size for stable sample with different concentrations of curcumin.

**Figure 3:**
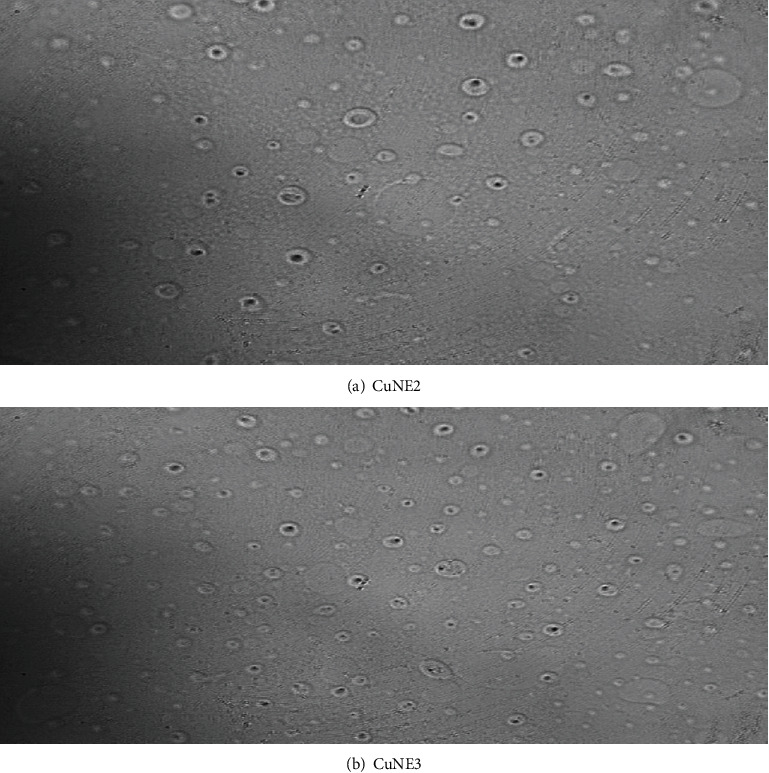
Optical image for curcumin encapsulation in different nanoemulsion samples.

Ahmed et al. [5] reported that stable nanoemulsion with 50 : 50 (LCT:SCT) oil ratio has an average droplet size of 200 nm and polydispersity less than 0.4. CNE with palm oil MCT (medium-chain triglyceride) has less water solubility than SCT (short-chain triglyceride) oil showing a polydispersity index in the range 0.3-0.357. The dynamic light scattering principle mean diameter size of nanoemulsion and its polydispersity index (PDI) was analyzed using the Nanotrac software. Table 2 shows data for stable nanoemulsion in terms of particle size and PDI, whereas Figures 1 and 2 show particle size distribution for stable and different concentrations of curcumin, respectively. Stable nanoemulsions are showing narrow particle size distribution with PDI less than 0.4. Ahmed et al. [5] reported that if PDI is higher than 0.5, emulsion would not be stable for a longer time. They also suggested that medium-chain triglyceride-based nanoemulsion provides better stability and enhances curcumin's bioaccessibility in terms of their release phenomenon. Figures 3(a) and 3(b) show the optical microscope image of CuNE with concentrations of 0.75 and 0.15 g curcumin. As shown in Figure 3, we can observe the perfect encapsulation of curcumin in oil-in-water morphology. Curcumin particles surrounded the layer of oil droplets in stable form without any aggregation. These oil droplets were tiny and dispersed thoroughly in nanoemulsion.

The total phenolic content of the nanoemulsion was used to calculate encapsulation efficiency. The Folin-Ciocalteu reagent was used to determine the total phenolic content of a stable nanoemulsion. The total phenolic content was determined before and after 30 mins of centrifugation at 5000 rpm.

This method used the standard calibration curve of gallic acid at different concentrations with *R*^2^ value of 0.9894 to calculate encapsulation efficiency. CuNE has a higher encapsulation efficiency because of the low surface tension between oil droplet flocculation, and reaggregation was avoided to improve curcumin solubility. As seen in the optimization of sonication time, polydispersity provides information about the homogeneity of the distribution of sizes, indicating the formation of monodisperse systems. According to this system, sonication would improve encapsulation efficiency. If the concentration of Tween 80 increases curcumin stability, then it also increases the reflection in encapsulation efficiency [22].

### 4.3. Curcumin Nanoemulsion in Bread Formulation and Its Analysis

Based on the above result, we took curcumin nanoemulsion (CuNE 2) for further study in terms of its addition in bread formulation.

### 4.4. Sensory Analysis of Bread

**Figure 4:**
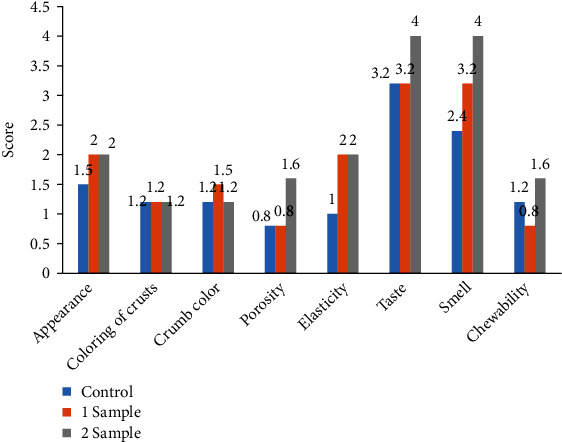
The results of the tasting evaluation of prototypes of bread with given weighting coefficients.

The total scoring study was formed based on quality indicators of bread such as appearance, character porosity, elasticity, crumb color, flavor, and mouthfeel of the crumb. For sensory analysis, age group with 25-50-year-old people was selected based on their evaluation point for each factor. As the significant color changes, the crusts of the samples have been noted and the indicators were mentioned earlier. The results of the tasting evaluation of bread are shown in Figure 4.

The evaluation results reveal that the sample of bread with the addition of a nanoemulsion based on curcumin (sample 2) has a considerably higher score than the control sample (control) and sample 1. Addition of curcumin nanoemulsion does not impart bad effect on organoleptic properties such as appearance, color of crust and crumb, and even chewability had even score as control sample. Bread sample containing curcumin nanoemulsion have better taste and smell than control sample and elasiticity of bread is more than control sample.

**Figure 5:**
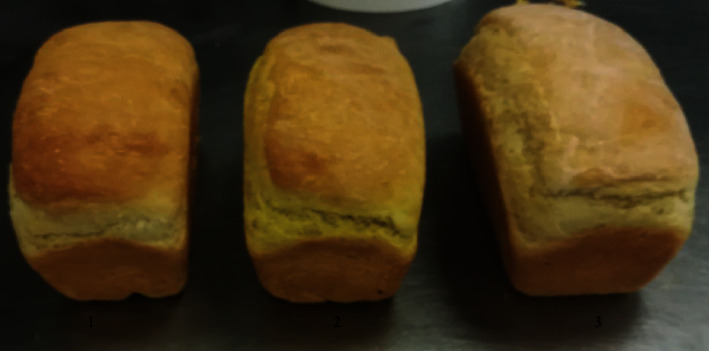
Photo of prototype bread (1: control; 2: sample 1; and 3: sample 2).

The indicator “character porosity” in bread sample has no seals and cavities, and sufficiently, uniform pores are small, medium-sized, and thin-walled compared to the control sample. Indicator “flavor” is pronounced, intense bouquet, well-baked bread from the well-attenuated test. As shown in Figure 5, color did not find any difference in all samples, where elasticity of samples 1 and 2 was better than the control sample. Taste, smell, and chewability of sample 2 are better than the control and sample 1; this is because of proper dispersion of nanoemulsion in bread formulation.

Thus, we can conclude that the nanoemulsion can improve the sensory quality parameters of bread, thereby increasing the attractiveness of the product for the consumer.

### 4.5. Physicochemical Parameters for Bread

The test samples of bread (control and samples 1 and 2) have investigated the physicochemical parameters of quality, such as humidity, acidity, and porosity (Table 3). The data obtained is shown in Table 3, allowing us to predict that curcumin nanoemulsion (sample 2) has a positive effect on the specific volume of the bread. The use of nanoemulsion provides structural and quantitative changes in porosity index of bread. In this sample, the crumb pores become more uniform and thin-walled. By porosity, the pore volume is a percentage of the total volume of crumbs of bread. The highest rate is observed in the sample with the addition of a nanoemulsion based on curcumin (sample 2) and is at a level of 69.4%. This sample would be better digested by the digestive juices and more digestible due to uniform porosity. Nanoemulsion improves the cell structure of bread with minor roughness and pore size. The lowest rate was recorded in the test piece of bread (control) -56.02.

Humidity in the crumb of the sample is much lower than the control sample (control) and sample bread with the addition of pulverized dry turmeric root (sample 1). When humidity of sample is less than 30%, it provide deteriorate flavor and increse hardness of bread sample immediately. When humidity exceeds 45%, it deteriorates the quality of the bread and reduced calorie content. Curcumin nanoemulsion shows 30.83% humidity, indicating that bread changes its taste but retains the quality in terms of shelf life and calorie content. Changes in the acidity had no significant difference from the samples (control and sample 1).

### 4.6. Scanning Electron Microscope Analysis of Bread

**Figure 6:**
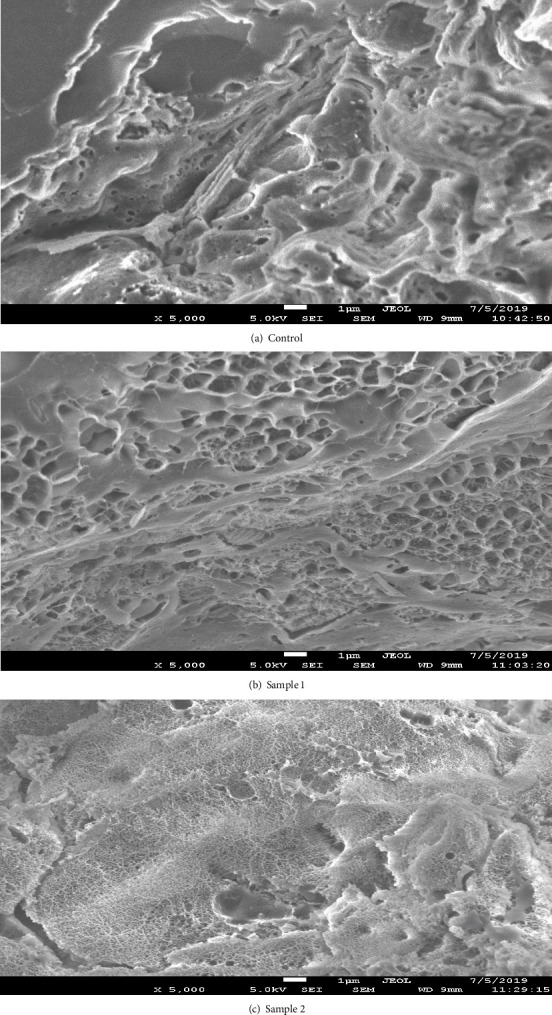
Photo study experienced crumb samples prepared using scanning electron microscopy (total magnification of ×5,000). Grain matrix: (a) control, (b) with the addition of crushed dry turmeric root, and (c) with the addition of nanoemulsions based on curcuminoids curcumin.

To study the flow of colloidal and biological processes as described in the SEM method section, samples were analyzed. The obtained photograph crumb sample is shown in Figure 6.

Based on the three-dimensional images, the control sample was observed with the presence of dense morphological structure without developing the grain matrix. In a sample of bread produced from a part of crushed dried root of turmeric, large size pores with few sealed nonporous sites were observed. An average thickness of pore wall is not uniform over all the surface of the crumb. In a sample of bread produced using a part of a nanoemulsion based on curcuminoids, curcumin was observed with a large number of tiny uniform pores. The presence of voids and seals is not established. This has the best structural characteristics of bread with nanoemulsions based on curcuminoids curcumin thus having an increased digestibility.

**Figure 7:**
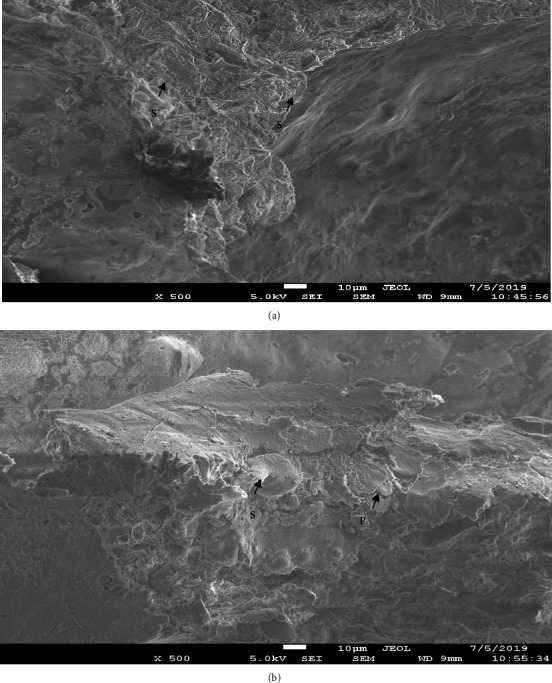
Photo study experienced crumb samples prepared using scanning electron microscopy (total magnification is ×500). Grain matrix: (a) control and (b) with the addition of crushed dry turmeric root (S: corn starch; P: protein matrix).

The control sample of bread (control) and in the bread with the addition of pulverized dry turmeric root (sample 1) was revealed with the presence of elongated and rounded starch grains surrounded by a matrix protein (Figure 7).

### 4.7. Deformation of Bread Sample

Changes experienced in dough rheology and crumb samples depend upon the insertion of additives as shown in Table 4. Deformation test changes and crumb of bread prototypes suggest a significant effect of the introduction of additives on the rheological characteristics of dough and bread crumbs. The dough is prepared based on dry milled turmeric root which has a higher performance than the samples (control and sample 2). Adding particulate dry turmeric root reduces the crumb's overall deformation, thereby increasing kroshlivosti. Adding to the composition of bread nanoemulsion based on curcuminoids, curcumin has a positive effect increasing the total strain and the elasticity of the crumb of bread.

### 4.8. Antioxidant Activity of Curcumin Nanoemulsion

Antioxidant capacity of control sample, sample 1, and sample 2 was expressed as *μ*g of trolox equivalents (TE) per g of nanoemulsion as shown in Table 5. In general, the nanoemulsion antioxidant capacity measured by FRAP assay did not present significant differences regardless of surfactant concentration. However, the antioxidant capacity values obtained by FRAP assay were significantly lower than by DPPH. In this regard, if some species are reduced, others have to be oxidized. According to Abbas et al. [23], the three active sites of curcumin can suffer oxidation by electron transfer and hydrogen abstraction. Test prototypes differed bread antioxidant activity not only depending on technological factors but also on the additives introduced into the formulation that is designed. Enriched bread test samples (samples 1 and 2) compared with a control sample (control) had a higher antioxidant activity for both ABTS and DPPH, as shown in Table 5. The prototype bread with the addition of a nanoemulsion based on curcuminoids curcumin (sample 2) showed the most significant antioxidant activity (2.3 times relative to the control sample). These results suggest a positive impact of the introduced additive in bread recipes developed. Nanoemulsion contributes to maintaining and enhancing BAS (curcumin) encapsulated in the nanoemulsion of the “oil-water” with sonication. This is because nanoemulsions containing Tween 80 antioxidant capacities varied regarding the surfactant concentration and depending on the scavenging assay for curcumin determination used. Although based on our results, high-concentration Tween 80 led to a slow release of curcumin, and it has been reported that an excess of this surfactant can form micelles able to entrap the bioactive compound within, which enhances the protection of encapsulated curcumin, thus increasing the antioxidant capacity of the system. One of the primer reason for maxium antioxidant of curucmin nanoemulsion is due to sonicatio parameter which embedded curcumin encapsulation in nanoemulsion and controlled its solubility in it.

### 4.9. Microbial Analysis of Curcumin Nanoemulsion Bread Samples

**Figure 8:**
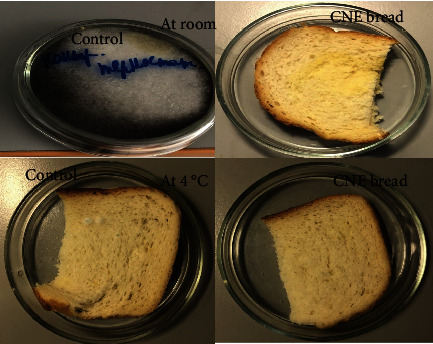
Microbial growth on bread sample during room temperature and refrigerator after 7 days.

Figure 8 shows curcumin nanoemulsion bread samples after placing at different two conditions for 7 days in comparison with control sample. It is found that at room temperature, control bread samples have black algae formed on its surface whereas nanoemulsion sample is still yet to form any kinds of algae on its surface. Same observation was found at cold temperature; compared to nanoemulsion bread sample, control sample found growth of algae. Even after stored for 14 days, nanoemulsion sample did not show any kind of growth of algae. This is because nanoemulsion sample contains curcumin which inhibits the growth of algae, as curcumin contains polyphenol compound which acts as antioxidant properties.

## 5. Conclusion

We conclude that our current work provides the successful formation of stable curcumin encapsulation in nanoemulsion with a high percent of loading (0.15 g) containing Tween 80 surfactant using ultrasound approach and its incorporation in bread formulation. Based on the data, we can conclude that the positive impact of a nanoemulsion based on curcuminoids curcumin in baked goods technology is appropriate, as it allows to increase the antioxidant activity of bread. Entering the additive composition has a positive effect on the organoleptic and physicochemical indicators of bread quality. In addition, it allows you to expand the range of products of mass consumption curative properties. It is worth noting that introducing the nanoemulsion of the test results in a reduction of total deformation after test obminki; more research is needed. Bread, obtained using the nanoemulsion, has a porosity developed compared to control sample and sample 1.

## Figures and Tables

**Table 1:** Formulation prototype wheat bread.

Ingredients	The amount of raw materials for 1 kg of flour		
	The control (g)	Sample 1 (g)	Sample 2 (g)
1. Flour	580	580	580
2. Pressed yeast	11	11	11
3. Sugar	16.6	16.6	16.6
4. Salt	11	11	11
5. Margarine	20.75	20.75	—
6. Dry chopped turmeric root	—	1	—
7. The nanoemulsion	—	—	20.75
8. Water	360.6	360.6	360.6
Total	1000	1000	1000

**Table 2:** pH and gravitational stability of nanoemulsion data.

Exp no.	pH	Gravitational stability at room temperature (days)	Particle size for fresh sample (nm)	Particle size after 60 days (nm)	PDI	Zeta potential (mV)	Encapsulation efficiency
CuNE 1	5.72	10	140 ± 05	720 ± 5	0.63	-15	95%
CuNE 2	5.72	60	13.35 ± 0.5	14.05 ± 0.5	0.27	-25	
CuNE 3	5.74	65	11.05 ± 05	12 ± 05	0.35	-27	
CuNE 4	5.33	15	115 ± 3	625 ± 10	0.43	-18	

**Table 3:** Physical and chemical properties of prototypes of bread.

Indicator	Norm GOST 27842-88	The control	Sample 1	Sample 2
Humidity (%)	Not more than 45.0	41.97 ± 0.5	40.69 ± 0.5	30.83 ± 0.5
Acidity (o)	Not more than 3.0	1.7 ± 0.1	1.8 ± 0.1	1.8 ± 0.1
Porosity (%)	Not less than 68.0	56.02 ± 1.0	61.7 ± 1.0	69.4 ± 1.0

**Table 4:** The rheological characteristics of dough and crumb of bread prototypes.

Specimen	Total deformation (h tot) (mm)	Plastic deformation (h mp) (mm)	Elastic deformation (h simp) (mm)	The elasticity of the crumb (.∆*h*)
Deformation characteristic test (after obminki)				
The control	30.233 ± 1.2	27.963 ± 0.55	2.270 ± 0.19	0.075 ± 0.023
Sample 1	48.868 ± 0.5	44.125 ± 0.5	4.743 ± 0.2	0.097 ± 0.013
Sample 2	29.266 ± 0.7	27.386 ± 0.477	1.880 ± 0.14	0.064 ± 0.01
Deformation characteristics of the crumb				
The control	6.250 ± 0.23	1.822 ± 0.33	4.428 ± 0.18	0.708 ± 0.01
Sample 1	6.232 ± 0.28	2.106 ± 0.15	4.126 ± 0.21	0.662 ± 0.01
Sample 2	6.250 ± 0.27	2.202 ± 0.19	4.048 ± 0.16	0.647 ± 0.01

**Table 5:** Antioxidant activity in terms of DPPH and ABTS.

Specimen	DPPH (% inhibition)	ABTS (% inhibition)
The control	20.59 ± 0.05	22.75 ± 0.15
Sample 1	27.64 ± 0.015	28.5 ± 0.05
Sample 2	31.29 ± 0.02	32.11 ± 0.1

Note: all analysis measurement was done in triplets and has ± SEM (*p* < 0.05).

**Table 6:** Experimental setup for curcumin nanoemulsion.

Exp no.	Palm oil (g)	Tween 80 (g)	Curcumin concentration (g)	Water (g)	Stability
CuNE 1	2.4	0.2	0.15	95.45	Unstable
CuNE 2	2.4	0.6	0.15	91.50	Stable
CuNE 3	2.4	0.6	0.075	91.55	Stable
CuNE 4	3.4	0.6	0.15	90.5	Unstable

## Data Availability

The datasets generated for this study are available on request to the corresponding author.
